# Green Schoolyards in Low-Income Urban Neighborhoods: Natural Spaces for Positive Youth Development Outcomes

**DOI:** 10.3389/fpsyg.2018.00805

**Published:** 2018-05-25

**Authors:** Carolyn R. Bates, Amy M. Bohnert, Dana E. Gerstein

**Affiliations:** ^1^Department of Psychology, Loyola University Chicago, Chicago, IL, United States; ^2^Nutrition Policy Institute, University of California Agriculture and Natural Resources, Berkeley, CA, United States

**Keywords:** green space, schoolyards, urban, child development, prosocial behavior, physical activity

## Abstract

Children from low-income families are increasingly growing up in urban areas with limited access to nature. In these environments, strategies that promote access to natural outdoor spaces, such as green schoolyards, may enhance positive youth development outcomes by promoting physical activity (PA) and prosocial behavior, as well as increasing perceptions of safety. The current study examines children’s PA and social interactions, as well as caregiver and teacher perceptions of safety, injuries, teasing/bullying, and gang activity on three newly renovated green schoolyards in low-income urban neighborhoods. A multi-method strategy, including behavioral mapping and caregiver- and teacher-reported surveys, was utilized at three time points to examine positive youth development outcomes and maintenance of effects over time. Analyses revealed that children evidenced a range of PA on the green schoolyards and demonstrated significant decreases in sedentary activity over time. The majority of children were engaged in social interactions with peers on the green schoolyards when observed. Less than 3% of interactions were negative and follow-up analyses found significant increases in positive interactions on the green schoolyards up to 24 months post-renovation. Caregivers and teachers reported increased perceptions of safety, fewer injuries, less teasing/bullying, and less gang-related activity on the renovated green schoolyards in comparison to the pre-renovation schoolyards, and these effects were maintained up to 32 months post-renovation. Overall, the study suggests that green schoolyards may promote positive development outcomes among youth living in urban, low-income neighborhoods by providing natural and safe spaces for PA and prosocial behavior.

## Introduction

Rates of urbanization have reached unprecedented levels with over half of the world’s population living in urban areas. This number is expected to continue to climb, resulting in more than two thirds of people living in urban settings by 2050 (World Health Organization [WHO], 2010; United Nations Department of Economic and Social Affairs Population Division, 2014). Although urbanization has been associated with important economic and social transformations, other effects such as increased crowding, industry, and infrastructure can lead to individual and societal problems, including higher rates of crime and limited access to nature (Shelley, 1981; Frumkin, 2002). Further, urbanization has led to significant social and health inequities in low-income communities compared to more affluent communities (Mitchell and Popham, 2008). Although the specific effects of urbanization on children’s development are not well-understood, research suggests that children growing up in low-income urban environments with limited access to green space may have fewer opportunities to engage in positive behaviors, including physical activity (PA) (Gomez et al., 2004; Weir et al., 2006), and may be at risk for increased rates of behavior problems, including oppositional and conduct disorders (Leventhal and Brooks-Gunn, 2000; Manly et al., 2013; Markevych et al., 2014). Research suggests that increased access to nature may buffer these effects by promoting positive development outcomes among urban youth (Wells, 2000; Wells and Evans, 2003), and may reduce health inequalities in low-income communities (Mitchell and Popham, 2008).

Two recent reviews evaluated literature examining the impact of nature on health and well-being in urban settings (Haluza et al., 2014; Gascon et al., 2015). Haluza et al. (2014) conducted a narrative review (*N* = 17 studies) of nature’s impact on physiological outcomes (i.e., brain activity, cardiovascular system, endocrine system, and immune functioning) in college students and adults and concluded that exposure to nature had a harmonizing effect on physiological stress reactions across body systems. However, most of the reviewed studies were cross-sectional, highlighting the need for longitudinal research examining exposure to nature over time. Gascon et al. (2015) systematically reviewed the long-term mental health benefits of residential green and blue spaces (*N* = 28 studies) and concluded that there was evidence for a causal relation between surrounding greenness and mental health in adults, but the data were less conclusive in child samples. In summary, these studies highlight the need for additional work to examine the impact of exposure to nature on physical and mental health in urban settings, particularly among children.

One way to examine the effects of nature among children in urban areas is through studies of renovated green schoolyards. Green schoolyards are multi-purpose, environmentally beneficial spaces that incorporate natural elements, such as gardens, wooded areas, and green spaces, with traditional play features, and often include outdoor classrooms or learning components as well (Plovnik and Strongin, 2015; Healthy Schools Campaign and Openlands, 2016). Bell and Dyment (2008) narratively reviewed literature examining the impact of green schoolyards on physical, mental, social, and spiritual health of students. The authors concluded that preliminary evidence for such relations was promising, but that studies had been largely exploratory to that point and that the field was in need of additional work utilizing more sophisticated study designs for substantiation.

Several subsequent studies have implemented greater methodological rigor to examine the impact of schoolyards on positive developmental outcomes in youth. By providing natural spaces for activity, schoolyards may reduce daily sedentary behavior and promote PA, both of which have been associated with positive physical and developmental outcomes among youth (Janssen and LeBlanc, 2010; Tremblay et al., 2011). Brink et al. (2010) and Anthamatten et al. (2011) used a quasi-experimental design and the System for Observing Play and Leisure Activity in Youth (SOPLAY) observation methodology to examine elementary school children’s utilization and PA on six renovated green schoolyards and three non-renovated schoolyards in Denver, CO, United States. Across both studies, renovated green schoolyards were more highly utilized than non-renovated schoolyards. Brink et al. (2010) observed that students at renovated schools had higher overall levels of activity, regardless of when the schoolyard was renovated (e.g., 1- or 2-years prior to the evaluation). Upon further examining PA in the same sample, Anthamatten et al. (2011) found that the percentage of students who were active on the green schoolyards was not significantly different between renovated and non-renovated schoolyards. Andersen et al. (2015) examined children’s PA in the context of differing surface materials within green schoolyards among a sample of Danish children ages 10–15. The study observed that children engaged in higher levels of PA on the grass and playground areas of the schoolyard, in comparison to the blacktop or hard surface areas (Andersen et al., 2015). Finally, in another quasi-experimental study, Cohen et al. (2015) used the System for Observing Play and Recreation in Communities (SOPARC) observation methodology and surveys to assess pre- and post-renovation utilization, energy expenditure, and perceptions of safety at four community parks in San Francisco, CA, United States (i.e., two non-renovated and two undergoing renovation). In comparison to the non-renovated parks, coders observed greater utilization of the renovated parks and higher overall energy expenditure by users at the renovated parks. Based on analysis with the baseline and follow-up data, users from the renovated parks reported significant increases in perceptions of park safety, which may have positively impacted utilization and activity levels (Cohen et al., 2015). To summarize, several studies have established that renovated parks and green schoolyards promote increased utilization and may support positive PA outcomes. However, few studies have followed these effects longitudinally and in predominantly low-income urban communities to examine the maintenance of schoolyard utilization and PA over time.

In addition to promoting positive physical development outcomes through increased utilization and PA, renovated green schoolyards may encourage prosocial behaviors among youth. Chawla et al. (2014) conducted interviews and ethnographic observations of early elementary through high school students on a variety of schoolyards across urban and suburban settings to examine the impact of nature on socio-emotional well-being. Students at schools with renovated green schoolyards demonstrated prosocial behaviors (e.g., forming supportive groups) in addition to low levels of stress, anger, and problem behaviors (Chawla et al., 2014). In a quasi-experimental study, Carrus et al. (2015) observed 39 Italian preschool children (ages 18 months–3 years) attending green or non-green daycare centers. Children at green daycare centers displayed more positive affect and prosocial interactions than children at non-green daycare centers, but only after free play in outdoor green spaces. Thus, the authors concluded that contact with nature may promote more positive affect and social interactions among youth (Carrus et al., 2015). In a quantitative cross-sectional study that surveyed 172 urban children from Spain, Corraliza et al. (2012) found that children who reported having greater access to nature in the home and school settings (e.g., green school grounds, neighborhood green spaces, and views of nature through windows) also reported lower levels of perceived stress than children with lower access to nature, despite reporting similar exposure to adversity. Additionally, exposure to nature buffered the association between reported adversity and perceived stress, and authors suggested that exposure to nature may have promoted positive coping (Corraliza et al., 2012). Another cross-sectional study of green space and stress among 10-year-old German children found that children living within 500 m of an urban green space had fewer parent-reported behavior problems than children living a greater distance from green space. When stratified by sex, the result was only significant among males (Markevych et al., 2014). Together, these studies provide preliminary evidence for associations between exposure to nature and positive social development, including prosocial behaviors, but more rigorous methodologies are needed to confirm these associations as well as considering the benefits among youth living in low-income urban neighborhoods.

Renovating green schoolyards in low-income urban neighborhoods may impact perceptions of safety in these areas, and this may be beneficial to positive development by supporting schoolyard utilization, PA, and prosocial behavior. A study by Farley et al. (2007) demonstrated that providing safe schoolyard settings in urban areas resulted in increased PA and decreased sedentary behavior among youth in that neighborhood. Similarly, a recent study showed that among renovated urban schoolyards, those perceived to be clean and safe receive the greatest amount of utilization and PA by children and adults (Colabianchi et al., 2011). In Chawla et al. (2014) ethnographic study, students attending a school with a green schoolyard reported that this natural space was a haven from teasing and bullying that occurred inside the school walls, and this coincided with positive social-emotional outcomes at these schools. Increased perceptions of safety may also support schoolyard utilization, PA, and positive social outcomes. Indeed, these studies highlight that green schoolyard renovations may impact several components of safety, including the overall condition of the schoolyard (e.g., risk of injury during play), the surrounding community (e.g., gang activity), and student interactions (e.g., teasing and bullying), and each of these may enable children to best utilize and benefit from green schoolyard renovations. Parents and teachers’ perceptions of schoolyard safety may be of particular importance for student’s positive development because of parent and teacher influence on access and utilization. Children’s access to schoolyards during the school day is dependent on teacher’s utilization of the schoolyard for recess, physical education, and classroom instruction. During outside of school time, children’s access to the schoolyard is influenced by parental rules around traveling to and utilizing the schoolyard. Therefore, it is important to assess not only children’s positive development outcomes in the context of renovated schoolyards, but also parent and teacher perceptions of the safety, which may provide indications of whether schoolyards are being utilized to their greatest potential, or if there are remaining barriers.

The current study aims to expand the literature by examining the impact of nature, specifically renovated green schoolyards, on children’s positive development outcomes over time in the context of low-income urban neighborhoods. The study considers two positive development outcomes – PA and social interactions – as well as perceptions of student safety, injuries, bullying, and gang activity in the context of three recently renovated green schoolyards and examines the longitudinal course of these outcomes to investigate the maintenance of effects. The effects of age, gender, and race/ethnicity are examined to understand whether positive development outcomes in the context of green schoolyards differs among various subgroups. The study aims to contribute a novel perspective on the benefits of green schoolyards on youth growing up in low-income urban neighborhoods and support the prioritization of green infrastructure in high-density urban areas.

## Materials and Methods

### Schools and Participants

**FIGURE 1:**
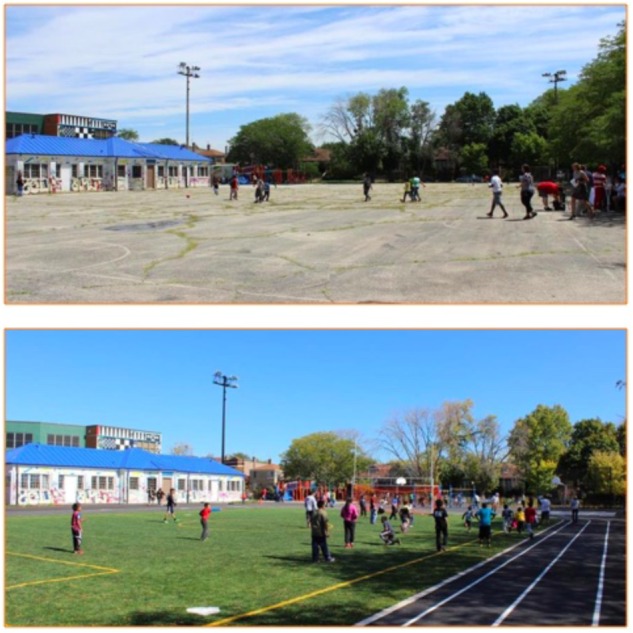
Before and after photos of schoolyard renovation at School 1. Photos courtesy of Space to Grow.

**FIGURE 2:**
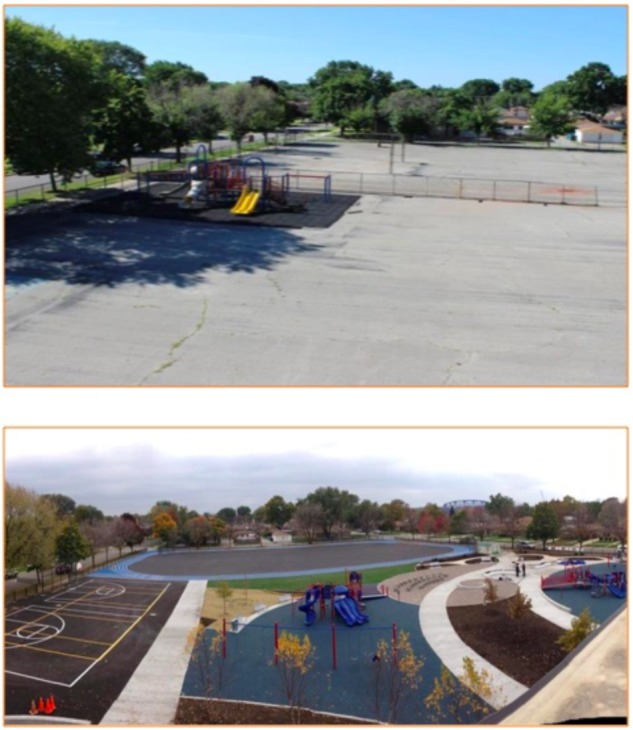
Before and after photos of schoolyard renovation at School 2. Photos courtesy of Space to Grow.

**FIGURE 3:**
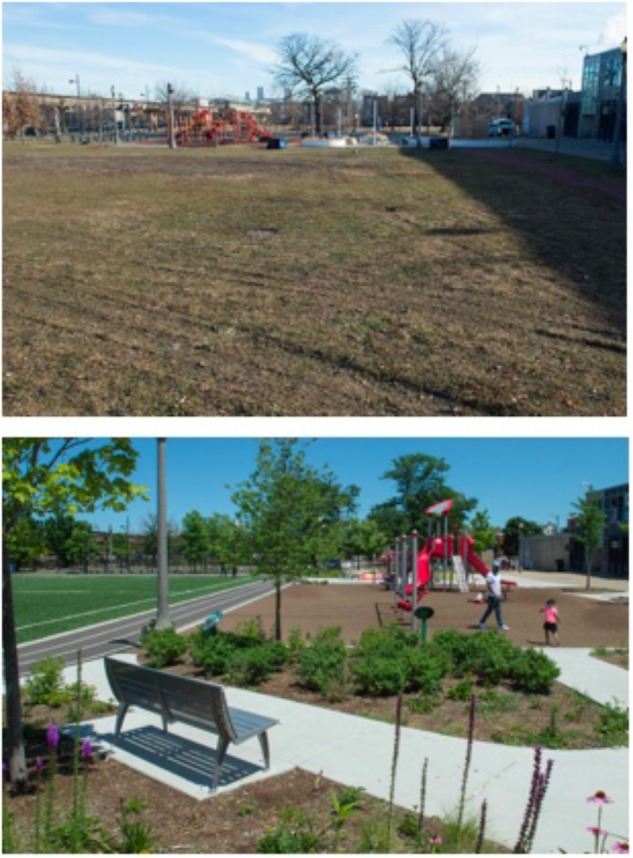
Before and after photos of schoolyard renovation at School 3. Photos courtesy of Space to Grow.

Data were collected at three public elementary schools in Chicago, IL, United States that had recently undergone a green schoolyard renovation through the Space to Grow (STG) initiative (see **Figures 1–3**). STG is a multi-sector, public-private partnership managed by Healthy Schools Campaign and Openlands (two non-governmental organizations) that seeks to support health, education, and a connection with nature in underserved urban communities across Chicago by renovating schoolyards to meet the needs of the respective schools and communities (Healthy Schools Campaign and Openlands, 2016). Schools were pre-selected by the capital and managing partners and invited to apply. Schools were required to demonstrate two key needs: (1) a surrounding community that lacked access to safe, well-maintained green space, and (2) community issues with storm water control and flooding. The application was completed by school staff and required demonstrating support from the Local School Council and alderman. Each school was also required to commit to keeping the schoolyard open to the public and to maintain the space following the renovation. Each STG school community took part in a planning process during which school staff, students, caregivers, and other community members provided a vision for their schoolyard through open houses and planning meetings held at the schools. Based on the input gathered at these meetings, the schoolyards were designed and constructed to meet the unique needs and visions of each community. The three schools in the current study were located in three distinct neighborhoods across the south and west sides of Chicago and enroll a high percentage of low-income, minority students (see **Table 1**). All schools enrolled children from pre-kindergarten through eighth grade. Two schools were renovated during the summer of 2014, and one school was renovated during the summer of 2015.

**Table 1:** Demographics of study schools.

	School 1	School 2	School 3
Neighborhood	Chicago Lawn	Hegewisch	East Garfield Park
Student population	753	368	397
Demographics	39% Black, 60% Hispanic, 1% White	76% Hispanic, 18% White, 5% Black, 1% Other	97% Black, 2% Hispanic, 1% Other
Low income students^∗^	96%	58%	90%
Date of schoolyard renovation	Summer 2014	Summer 2014	Fall 2015

*^∗^Low-income status is measured by eligibility to participate in free or reduced-price school meal program.*

Data were collected using a multi-method procedure, which included observational assessments and survey administration. Observational data were collected on two schooldays and one weekend day at each time point. Schoolyard observations (total observations *N* = 7,025) occurred during the spring and fall of 2016 (i.e., T1 and T2; see **Table 2**), and surveys were collected during the spring of 2016 and spring of 2017 (i.e., T1 and T3; see **Table 2**). Observational data were collected 6-months apart (i.e., in spring and fall) to allow for variation based on seasonality, whereas survey data were collected 1-year apart to provide a robust longitudinal examination of differences in perceptions of safety. Staffing needs and minimizing study burden were also major determinants of data collection timing. The institutional review board of Loyola University Chicago, the University of California, Davis, and the research review board of Chicago Public Schools approved all study procedures.

**Table 2:** Study time points and measurement strategies.

	Time 1 (Spring 2016)	Time 2 (Fall 2016)	Time 3 (Spring 2017)
Behavioral mapping *(i.e., physical activity and social interactions)*	X	X	
Surveys *(i.e., perceptions of safety, injuries, teasing/bullying, and gang activity)*	X		X

### Measures

#### Behavioral Mapping

Children’s behaviors on the schoolyard were objectively assessed using behavioral mapping methodology (Cosco et al., 2010, 2014). Behavioral mapping is a design-sensitive measurement system allowing for the objective observation of PA and associated schoolyard components and attributes. Using this approach, each renovated schoolyard was divided in advance into 10–14 observation zones with the purpose of providing data collectors with a designated space that was easily scanned from a specified observation point. Two data collectors simultaneously followed a prescribed sequence of observation zones to circumnavigate the schoolyards. Data collectors employed a visual scanning protocol for each observation zone in a clockwise direction from the designated observation point, observing a single individual at a time. Upon observation of an individual, the data collector recorded the location of the person on a map of the schoolyard using GIS technology, and immediately recorded observational information on each variable of interest relating to that individual. The data collector then returned to the scanning protocol to collect information on other individuals in the observation zone (Cosco et al., 2010). Because behavioral mapping observes a single individual at a time, the amount of time that data collectors spent in each observation zone varied based on the number of individuals in the zone, as did the amount of time spent on a full circulation of the schoolyard.

Data collectors used behavioral mapping to code PA using the Child Activity Rating Scale (DuRant et al., 1993), which categorizes level of PA on an objective five-point scale: (1) stationary/motionless, (2) stationary with movement of limb(s) or very easy movement of trunk, (3) translocation (slow speed/easy), (4) translocation (medium speed/moderate), (5) translocation (fast or very fast/hard). Observations of social interactions used codes from the System for Observing Children’s Activity and Relationships during Play (SOCARP; Ridgers et al., 2010), which included categorizing observed interactions as positive (e.g., smile, high five, hug, positive statement to another individual), negative (e.g., grimacing, fighting, shoving, negative statement to another individual), neutral (i.e., in contact with another individual but no observable physical or verbal sign of valence), or no social interaction (i.e., not interacting with another individual). Data collection occurred at specific times during the school day (i.e., before school, during recess, during gym, and after school) and on the weekends. Behavioral mapping was also used to record observable characteristics of persons utilizing the schoolyard space, including gender (i.e., male and female), and race/ethnicity (i.e., African–American, Latino, Caucasian, Asian, and unknown/other). The approximate age of persons utilizing the schoolyard was also coded. Because many classrooms/grades often utilized the schoolyard at the same time, age was coded in groupings: toddler/preschool, kindergarten-4^th^ grade, 5^th^–8^th^ grade, high school or adult. School recess and physical education class schedules were utilized to assist data collectors in accurately coding the age of children on the schoolyard.

#### Surveys

Caregivers and teachers retrospectively reported on changes in student safety, injuries, teasing/bullying, and gang activity following the green schoolyard renovation via self-administered surveys. Survey respondents were asked to report on safety, injuries, and teasing/bullying using the following prompts “In your opinion since the schoolyard was redesigned:

(i) has the safety of the schoolyard changed?
(ii) has the number of injuries on the schoolyard changed?
(iii) has the amount of teasing or bullying between students on the schoolyard changed?
(iv) has gang-related activity on the schoolyard (e.g., threats, bullying, and gang presence) changed?”

Survey respondents were given a Likert scale of five answer choices ranging from “much more [safe, injuries, teasing and bullying, or gang-related activity] to much less [safe, injuries, teasing and bullying, or gang-related activity].

### Analyses

Descriptive analyses, including means and standard deviations, were used to characterize study variables. Independent samples *t*-tests and analyses of variance (ANOVAs) were utilized to test for significant differences in PA between sub-groups of individuals observed (i.e., age, gender, and race/ethnicity), by time of day, and to evaluate significant changes in observed PA and reported student safety, injuries, teasing and bullying, and gang-related activity over time, whereas chi-square analyses were used to examine differences in social interactions by subgroups and examine significant changes in social interactions over time.

## Results

Children observed via behavioral mapping ranged in age from pre-k through 8^th^ grade (median = middle school). The majority of children observed were African/American (44.7%) or Latino/Hispanic (39.2%) and males (55%). Survey data were collected from teachers, administrators, and school staff (*n* = 33 at T1; *n* = 40 at T3) and caregivers (*n* = 64 at T1; *n* = 61 at T3). Only 23 of 97 T1 participants (24%) completed surveys at T3 (*n* = 9 caregivers; *n* = 14 teachers). As such, data were treated as independent samples for analytic purposes. Descriptive analyses showed a wide range of PA on the schoolyards. Nearly one-third of the children observed were engaged in light, moderate, or vigorous PA (e.g., walking or running), whereas another third were stationary with some upper or lower body movement (e.g., swinging, kicking, and throwing; **Table 3**). Sub-group analyses found a significant impact of age on PA [*F*(4, 3250) = 21.83, *p* < 0.001]. Specifically, children in grade k-4 were significantly more active than children in grades 5–8 (*p* < 0.01), and adults were less active than all other age groups on the schoolyard (*p* < 0.05). Analyses by gender revealed that males were more active than females on the schoolyards [*t*(3316) = 7.59, *p* < 0.001]. There were no significant differences in level of PA by ethnicity or by time of day. Follow-up analyses revealed that there was a significant increase in overall PA on the schoolyards over time [*t*(7024) = -2.84, *p* < 0.001]. The greatest change in PA between time points resulted from a decrease in children who were stationary/motionless from T1 to T2 coupled with an increase in children who were stationary with some limb or trunk movement (**Table 3**), indicating that children were less sedentary at T2 when compared to T1.

**Table 3:** Physical activity (PA) observed on renovated green schoolyards at T1 and T2.

	Stationary	Stationary w/limb movement	Light PA	Moderate PA	Vigorous PA
T1 (*n* = 3,345)	33.98%	29.41%	29.50%	6.31%	0.78%
T2 (*n* = 3,710)	21.71%	45.40%	29.12%	4.08%	0.68%

Regarding observed social interactions, 63% of children observed were interacting with others at T1, with approximately 33% engaged in neutral interactions, 27% engaged in positive interactions, and less than 3% engaged in negative interactions (**Table 4**). Females (*x*^2^= 11.85, *p* < 0.01), African–American children (*x*^2^= 15.18, *p* < 0.01), and children in grades 5–8 (*x*^2^= 29.11, *p* < 0.001) were more likely to be interacting with others on the schoolyard. There was a significant impact of ethnicity on the valance of social interactions (*x*^2^= 14.64, *p* < 0.01), with a greater proportion of negative interactions than expected among African–American and White children, and a greater proportion of positive interactions than expected among Latino/Hispanic children. There was also a significant impact of age on social interactions (*x*^2^= 17.56, *p* < 0.01), such that there was a greater proportion of positive interactions than expected among children in grade k-4, and a greater proportion of neutral and negative interactions than expected among children in grades 5–8 and adults. There was no significant impact of gender on the valence of observed social interactions. Additionally, there were significant changes in observed social interactions on the schoolyards over time (*x*^2^= 98.80, *p* < 0.001), such that a greater percentage of children were interacting socially with each other on the schoolyards at T2. Based on the observed social interaction data, a greater percentage of children were interacting with others on the schoolyard at T2 when compared with T1, with increases in positive and neutral interactions. Negative interactions remained stable at T2 when compared with T1 (**Table 4**).

**Table 4:** Social interactions observed on renovated green schoolyards at T1 and T2.

	Negative	Positive	Neutral	No interaction
T1 (*n* = 3,345)	2.80%	27.10%	32.70%	37.00%
T2 (*n* = 3,710)	2.50%	35.20%	35.60%	26.70%

Caregivers and teachers retrospectively reported that compared to pre-renovation, the schoolyards were safer, students experienced fewer injuries, and there was less teasing/bullying and gang-related activity on the schoolyards at T1 (**Table 5**). Analyses demonstrated that caregivers and teachers maintained these perceptions at 1-year follow-up, with no significant changes in reports from T1 to T3 (*p* > 0.05; **Table 5**).

**Table 5:** Reported changes in student safety, injuries, teasing/bullying, and gang activity following green schoolyard renovation.

	Caregivers			Teachers		
	T1 (*n* = 64)	T3 (*n* = 61)	*t*-Value	T1 (*n* = 33)	T3 (*n* = 40)	*t*-Value
Safety^1^	0.77	1.03	–1.39^∗^	1.24	1.21	0.15^∗^
Injuries^2^	0.80	0.90	–0.58^∗^	0.77	0.69	0.37^∗^
Teasing/bullying^3^	0.66	0.65	0.07^∗^	0.53	0.53	0.03^∗^
Gang-related activity^4^	0.68	0.86	–1.07^∗^	0.57	0.77	–0.83^∗^

*Significance of changes tested using independent samples *t*-tests.*

*^*1*^Scale: -2 = much less safe, -1 = less safe, 0 = no change, 1 = more safe, 2 = much more safe. ^*2*^Scale: -2 = many more injuries, -1 = more injuries, 0 = no change, 1 = fewer injuries, 2 = many fewer injuries. ^*3*^Scale: -2 = much more teasing/bullying, -1 = more teasing/bullying, 0 = no change, 1 = less teasing/bullying, 2 = much less teasing/bullying. ^*4*^Scale: -2 = much more gang-related activity, -1 = more gang-related activity, 0 = no change, 1 = less gang-related activity, 2 = much less gang-related activity.*

*^∗^Non-significant (*p* > 0.05).*

## Discussion

Given the increasingly high rates of global urbanization and potential impact on health, particularly among low-income communities, it is important to identify effective and scalable ways to promote positive development outcomes among youth in urban areas. Evidence suggests that green schoolyards may positively impact youth development outcomes including PA and prosocial interactions. The present study builds on current literature by examining systematic observations of PA and social interactions on renovated green schoolyards in urban, low-income neighborhoods, as well as the maintenance of these outcomes over time to evaluate the stability of effects.

Children observed in this study engaged in a wide range of activity on the renovated green schoolyards. The most frequent type of PA observed was stationary with some trunk or limb movement (e.g., kicking, throwing, or bending), which neither qualifies as sedentary time nor an episode of acute PA. However, studies have demonstrated that this type of movement, especially in the context of the school day, can interrupt bouts of sedentary time to mitigate physical health risks and promote classroom engagement (Saunders et al., 2013; Hinckson et al., 2016). Moreover, nearly one-third of children were moving when observed, and most of these children were observed to be engaging in light PA. The overall level of activity did not vary significantly between the before-, during-, and after-school, suggesting that schoolyards served as a resource for PA across the entire day when school was in session. Although pre- and post-renovation data was not available for the current study, evidence suggests that park renovations in underserved urban communities promote increased utilization and PA among community members (Tester and Baker, 2009; Cohen et al., 2015). Two recent reviews concluded that acute episodes of PA benefit children’s cognitive functioning, and may facilitate engagement, attention, and learning in the classroom (Lees and Hopkins, 2013; Donnelly et al., 2016). Additionally, PA may benefit psychosocial health, including mood and perceived competence (Sallis et al., 2000; Lees and Hopkins, 2013). Based on these findings, the use of the schoolyards throughout the entire school day is advisable in order to give students opportunities to interrupt sedentary time and participate in PA.

Consistent with national developmental trends in PA (Belcher et al., 2010), younger children (i.e., grade k-4) and males were more active on the schoolyard than older children (i.e., grades 5–8) and females. Studies have identified several possible reasons that PA may decrease during adolescence, including reduced social support for PA engagement, lower perceived athletic competence, and decreased access to organized activities (Bélanger et al., 2011). Although schoolyards provided space and opportunity for PA, additional strategies may be warranted to combat these developmental trends and encourage PA among older children and females. Structured before- and/or after-school programs have been shown to be effective in increasing PA in these groups (Mears and Jago, 2016), and may be useful to increase the effectiveness of green schoolyard interventions for promoting PA among older youth. One study by Black et al. (2015) found that although 50% of children were active on urban schoolyards in the absence of any programming, the percent of children who were active increased to 99% when participating in a structured walking program. Moreover, research has shown promising outcomes—including significant increases in PA—from structured community programming that specifically targets early adolescent urban minority girls (Bohnert et al., 2014, 2017), increasing both access to organized activities and social support among urban youth. Thus, future studies may consider testing organized activities as an adjunctive strategy to promote increased PA among higher risk groups of older adolescents and females, being attentive to promoting positive social support and increases in perceived athletic competence for optimal success.

Overall, students on the renovated green schoolyards engaged in high rates of positive or neutral social interactions, and very low rates of negative social interactions. This is promising given the context of the schools in urban, low-income neighborhoods. Other literature has suggested that green schoolyards may promote positive interactions among diverse samples of youth, perhaps by providing opportunities for diverse cooperative play, promoting positive affect (Chawla et al., 2014; Carrus et al., 2015), and increasing ability to cope with stress (Corraliza et al., 2012). Another promising finding in the current study was that there were no significant differences in negative interactions between males and females. Higher rates of negative social interactions among older students were observed though highlighting the need for continued social-emotional curriculum to facilitate positive social dynamics and problem-solving strategies among early adolescents. Higher rates of negative social interactions among African–American and White students as compared with Latino students may have been an artifact of the different types of activities engaged in on the schoolyards (e.g., structured and cooperative play, such as soccer, among Latino youth, versus unstructured play among African–American and White youth), but warrants further investigation.

Positive development through PA and prosocial interactions on green schoolyards can only occur when schoolyards are utilized by students. Studies have shown that utilization of renovated schoolyards can be negatively impacted by a number of factors, especially perceptions of safety in high-crime urban areas (Colabianchi et al., 2011). Thus, it was encouraging to find that caregivers and teachers perceived the renovated green schoolyards in the current study to be safer, with less teasing, bullying, and gang activity than the pre-renovation spaces. The positive caregiver and teacher perceptions observed in the current study may have been impacted by the STG community-engaged planning process, which focuses on engaging the caregivers, students, teachers, and community members in the design of the schoolyard with the goal of meeting the specific needs and desires of the school and surrounding community (Healthy Schools Campaign and Openlands, 2016). Indeed, more work is needed to understand the best practices for renovating schoolyards and other green spaces to facilitate community investment and utilization of the renovated space. Efforts to increase community cohesion and neighborhood safety may help to overcome barriers caused by negative perceptions and promote optimal benefits from built environment interventions.

A final encouraging result of the study was the overall maintenance of beneficial outcomes over time. Few studies have longitudinally assessed PA, social interactions, and changes in student safety, injuries, teasing/bullying, and gang-related activity in the context of renovated green schoolyards. Observational data from the current study demonstrated that the renovated green schoolyards were highly utilized throughout the school day at both time points. Results suggest that green schoolyards maintained their status as zones of PA and primarily positive social interactions over time, and showed decreases in sedentary time, as well as increases in overall social interactions and positive social interactions over time. Furthermore, caregiver and teacher reports of high levels of student safety, few injuries, and low levels of teasing/bullying and gang-related activity on the schoolyard did not change significantly over time, indicating that these positive perceptions remained stable up to 32-months post-schoolyard renovation.

The study is not without limitations, most notably that the current schoolyards were only assessed post-renovation, thereby precluding our ability to infer causality. The study examined green schoolyards in diverse low-income communities in Chicago that recently underwent renovations by the STG initiative, so results should be considered in this context and may not be generalize to other cities or other initiatives. Further, renovations involved updates to both green spaces and play facilities, which may have impacted results. Rigorous experimental studies are needed to understand the unique impact of added green space, play facilities, and structured programming to positive developmental outcomes. Seminal behavioral mapping literature notes that because this method focuses on coding within a predetermined setting rather than specific children, fast-moving children may not be coded if they vacated an observation zone before being coded, whereas stationary children may be coded more than once if they do not move between observation rounds (Cosco et al., 2010). We were not able to gather self-report data from students due to restrictions implemented by the school district and the timing of data collections. Finally, we were unable to examine perceived safety as a mechanism underlying schoolyard utilization and PA due to sampling strategy. Despite limitations, the study makes a unique contribution to the literature by being the first to longitudinally investigate positive development outcomes and perceptions of safety in the context of renovated green schoolyards in low-income urban neighborhoods.

The current study builds on existing literature that has shown benefits of green schoolyard renovations to PA, prosocial behavior, and safety, and provides additional evidence that renovated green schoolyards in low-income urban areas serve as a beneficial context of development for at risk youth. Furthermore, our study supports that the observed benefits of green schoolyards are maintained long-term, and that positive development outcomes on green schoolyards may even increase over time. Both PA and social interactions saw improvements over a 6-month period, up to 24 months post-renovation, and perceptions of safety remained stable over the course of a year, up to 32 months post-renovation. Taking these results in the context of other literature leads us to conclude that investing in built environments, particularly green schoolyards, may be an effective and enduring way to promote positive development outcomes among school-age youth, especially those living in low-income urban neighborhoods with limited other resources.

## Ethics Statement

This study was carried out in accordance with the recommendations of the Loyola University Institutional Review Board and the Chicago Public Schools Research Review Board. The protocol was approved by Loyola University of Chicago and Chicago Public Schools. All subjects gave written informed consent in accordance with the Declaration of Helsinki.

## Author Contributions

AB is the principal investigator of the current study and DG is a co-investigator. They designed the study and oversaw data collection, entry, analysis, and manuscript writing. CB is the project coordinator and managed data collection and entry, ran study analyses, and was the primary author of the manuscript.

## Conflict of Interest Statement

AB, DG, and CB were hired to conduct the Health and Wellness Evaluation of the Space to Grow Initiative by Healthy School Campaign.

## References

[B1] Andersen H. B. Klinker C. D. Toftager M. Pawlowski C. S. Schipperijn J. (2015). Objectively measured differences in physical activity in five types of schoolyard area. 134 83–92. 10.1016/j.landurbplan.2014.10.005

[B2] Anthamatten P. Brink L. Lampe S. Greenwood E. Kingston B. Nigg C. (2011). An assessment of schoolyard renovation strategies to encourage children’s physical activity. 8:27. 10.1186/1479-5868-8-27 21477325 PMC3094264

[B3] Bélanger M. Casey M. Cormier M. Filion A. L. Martin G. Aubut S. (2011). Maintenance and decline of physical activity during adolescence: insights from a qualitative study. 8:117. 10.1186/1479-5868-8-117 22017754 PMC3215642

[B4] Belcher B. R. Berrigan D. Dodd K. W. Emken B. A. Chou C. P. Spuijt-Metz D. (2010). Physical activity in US youth: impact of race/ethnicity, age, gender, & weight status. 42 2211–2221. 10.1249/MSS.0b013e3181e1fba9 21084930 PMC3242154

[B5] Bell A. C. Dyment J. E. (2008). Grounds for health: the intersection of green school grounds and health-promoting schools. 14 77–90. 10.1080/13504620701843426

[B6] Black I. E. Menzel N. N. Bungum T. J. (2015). The relationship among playground areas and physical activity levels in children. 29 156–168. 10.1016/j.pedhc.2014.10.001 25454386

[B7] Brink L. A. Nigg C. R. Lampe S. M Kingston B. A. Mootz A. L. van Vliet W. (2010). Influence of schoolyard renovations on children’s physical activity: the learning landscapes program. 100 1672–1678. 10.2105/AJPH.2009.178939 20634465 PMC2920958

[B8] Bohnert A. M. Bates C. R. Heard A. M. Burdette K. A. Ward A. K. Silton R. L. (2017). Improving urban minority girls’ health via community summer programming. 4 1237–1245. 10.1007/s40615-016-0333-x 28364374

[B9] Bohnert A. M. Ward A. K. Burdette K. A. Silton R. L. Dugas L. R. (2014). Active summers matter: evaluation of a community-based summertime program targeting obesogenic behaviors of low-income, ethnic minority girls. 2014 133–150. 10.1002/yd.20107 25530244

[B10] Carrus G. Passiatore Y. Pirchio S. Scopelliti M. (2015). Contact with nature in educational settings might help cognitive functioning and promote positive social behavior. 6 191–212. 10.1080/21711976.2015.1026079

[B11] Chawla L. Keena K. Pevec I. Stanley E. (2014). Green schoolyards as havens from stress and resources for resilience in childhood and adolescence. 28 1–13. 10.1016/j.healthplace.2014.03.001 24691122

[B12] Cohen D. A. Han B. Isacoff J. Shulaker B. Williamson S. Marsh T. (2015). Impact of park renovations on park use and park-based physical activity. 12 289–295. 10.1123/jpah.2013-0165 24956608 PMC4851467

[B13] Colabianchi N. Maslow A. L. Swayampakala K. (2011). Features and amenities of school playgrounds: a direct observation study of utilization and physical activity levels outside of school time. 8:32. 10.1186/1479-5868-8-32 21492455 PMC3094267

[B14] Corraliza J. A. Collado S. Bethelmy L. (2012). Nature as a moderator of stress in urban children. 38 253–263. 10.1016/j.sbspro.2012.03.347 21504673

[B15] Cosco N. G. Moore R. C. Islam M. Z. (2010). Behavior mapping: a method for linking preschool physical activity and outdoor design. 42 513–519. 10.1249/MSS.0b013e3181cea27a 20068497

[B16] Cosco N. G. Moore R. C. Smith W. R. (2014). Childcare outdoor renovation as a built environment health promotion strategy: evaluating the preventing obesity by design intervention. 28(Suppl. 3) S27–S32. 10.4278/ajhp.130430-QUAN-208 24380462

[B17] Donnelly J. E. Hillman C. H. Castelli D. Etnier J. L. Lee S. Tomporowski P. (2016). Physical activity, fitness, cognitive function, and academic achievement in children: a systematic review. 48 1197–1222. 10.1249/MSS.0000000000000901 27182986 PMC4874515

[B18] DuRant R. H. Baranowski T. Puhl J. Rhodes T. Davis H. Greaves K. A. (1993). Evaluation of the Children’s Activity Rating Scale (CARS) in young children. 25 1415–1421. 10.1249/00005768-199312000-00016 8107551

[B19] Farley T. A. Meriwether R. A. Baker E. T. Watkins L. T. Johnson C. C. Webber L. S. (2007). Safe play spaces to promote physical activity in inner-city children: results from a pilot study of an environmental intervention. 97 1625–1631. 10.2105/AJPH.2006.092692 17666701 PMC1963283

[B20] Frumkin H. (2002). Urban sprawl and public health. 117 201–217. 10.1016/S0033-3549(04)50155-3 PMC1497432 12432132

[B21] Gascon M. Triguero-Mas M. Martínez D. Dadvand P. Forns J. Plasència A. (2015). Mental health benefits of long-term exposure to residential green and blue spaces: a systematic review. 12 4354–4379. 10.3390/ijerph120404354 25913182 PMC4410252

[B22] Gomez J. E. Johnson B. A. Selva M. Sallis J. F. (2004). Violent crime and outdoor physical activity among inner-city youth. 39 876–881. 10.1016/j.ypmed.2004.03.019 15475019

[B23] Haluza D. Schönbauer R. Cervinka R. (2014). Green perspectives for public health: a narrative review on the physiological effects of experiencing outdoor nature. 11 5445–5461. 10.3390/ijerph110505445 24852391 PMC4053896

[B24] Healthy Schools Campaign and Openlands (2016). Available at: http://www.spacetogrowchicago.org/ [accessed January 04 2018].

[B25] Hinckson E. Salmon J. Benden M. Clemes S. A. Sudholz B. Barber S. E. (2016). Standing classrooms: research and lessons learned from around the world. 46 977–987. 10.1007/s40279-015-0436-2 26626071

[B26] Janssen I. LeBlanc A. G. (2010). Systematic review of the health benefits of physical activity and fitness in school-aged children and youth. 7:40. 10.1186/1479-5868-7-40 20459784 PMC2885312

[B27] Lees C. Hopkins J. (2013). Effect of aerobic exercise on cognition, academic achievement, and psychosocial function in children: a systematic review of randomized control trials. 10:E174. 10.5888/pcd10.130010 24157077 PMC3809922

[B28] Leventhal T. Brooks-Gunn J. (2000). The neighborhoods they live in: the effects of neighborhood residence on child and adolescent outcomes. 126 309–337. 10.1037/0033-2909.126.2.309 10748645

[B29] Manly J. T. Oshri A. Lynch M. Herzog M. Wortel S. (2013). Child neglect and the development of externalizing behavior problems: associations with maternal drug dependence and neighborhood crime. 18 17–29. 10.1177/1077559512464119 23136210 PMC3771700

[B30] Markevych I. Tiesler C. M. Fuertes E. Romanos M. Dadvand P. Nieuwenhuijsen M. J. (2014). Access to urban green spaces and behavioural problems in children: results from the GINIplus and LISAplus studies. 71 29–35. 10.1016/j.envint.2014.06.002 24953038

[B31] Mears R. Jago R. (2016). Effectiveness of after-school interventions at increasing moderate-to-vigorous physical activity levels in 5-to 18-year olds: a systematic review and meta-analysis. 50 1315–1324. 10.1136/bjsports-2015-094976 27222308

[B32] Mitchell R. Popham F. (2008). Effect of exposure to natural environment on health inequalities: an observational population study. 372 1655–1660. 10.1016/S0140-6736(08)61689-X 18994663

[B33] Plovnik A. Strongin F. (2015). Baden: Lars Müller Publishers.

[B34] Ridgers N. D. Stratton G. McKenzie T. L. (2010). Reliability and validity of the System for Observing Children’s Activity and Relationships during Play (SOCARP). 7 17–25. 10.1123/jpah.7.1.17 20231751

[B35] Sallis J. F. Prochaska J. J. Taylor W. C. (2000). A review of correlates of physical activity of children and adolescents. 32 963–975. 10.1097/00005768-200005000-00014 10795788

[B36] Saunders T. J. Tremblay M. S. Mathieu M. É. Henderson M. O’Loughlin J. Tremblay A. (2013). Associations of sedentary behavior, sedentary bouts and breaks in sedentary time with cardiometabolic risk in children with a family history of obesity. 8:e79143. 10.1371/journal.pone.0079143 24278117 PMC3835898

[B37] Shelley L. I. (1981). Carbondale, IL: Southern Illinois University Press.

[B38] Tester J. Baker R. (2009). Making the playfields even: evaluating the impact of an environmental intervention on park use and physical activity. 48 316–320. 10.1016/j.ypmed.2009.01.010 19463491

[B39] Tremblay M. S. LeBlanc A. G. Kho M. E. Saunders T. J. Larouche R. Colley R. C. (2011). Systematic review of sedentary behaviour and health indicators in school-aged children and youth. 8:98. 10.1186/1479-5868-8-98 21936895 PMC3186735

[B40] United Nations Department of Economic and Social Affairs Population Division. (2014). New York, NY: United Nations.

[B41] Weir L. A. Etelson D. Brand D. A. (2006). Parents’ perceptions of neighborhood safety and children’s physical activity. 43 212–217. 10.1016/j.ypmed.2006.03.024 16712912

[B42] Wells N. M. (2000). At home with nature: effects of “greenness” on children’s cognitive functioning. 32 775–795. 10.1177/00139160021972793

[B43] Wells N. M. Evans G. W. (2003). Nearby nature: a buffer of life stress among rural children. 35 311–330. 10.1177/0013916503035003001

[B44] World Health Organization [WHO] (2010). Urbanization and health. 88 245–246. 10.2471/blt.10.010410

